# A simple scoring algorithm based on intrinsic capacity for functional ability in community-dwelling older adults in Taiwan

**DOI:** 10.1186/s12877-024-04969-4

**Published:** 2024-04-25

**Authors:** Ya-Hui Chang, Chung-Ying Lin, Yu-Tsung Chou, Hung-Yu Chen, Hui-Chen Su, Yi-Lin Wu, Yi-Ching Yang, Wen-Hsuan Hou

**Affiliations:** 1 Department of Public Health, College of Medicine, National Cheng Kung University, Tainan, Taiwan; 2 Institute of Allied Health Sciences, College of Medicine, National Cheng Kung University, Tainan, Taiwan; 3 Department of Health Management Center, College of Medicine, National Cheng Kung University Hospital, National Cheng Kung University, Tainan, Taiwan; 4 Department of Family Medicine, College of Medicine, National Cheng Kung University Hospital, National Cheng Kung University, Tainan, Taiwan; 5 Department of Neurology, College of Medicine, National Cheng Kung University Hospital, National Cheng Kung University, Tainan, Taiwan; 6 Department of Nursing, College of Medicine, National Cheng Kung University Hospital, National Cheng Kung University, Tainan, Taiwan; 7 Department of Physical Medicine and Rehabilitation, School of Medicine, College of Medicine, Taipei Medical University, Taipei, Taiwan; 8 Department of Physical Medicine and Rehabilitation, Taipei Medical University Hospital, Taipei, Taiwan; 9 Cochrane Taiwan, Taipei Medical University, Taipei, Taiwan

**Keywords:** Intrinsic capacity, Integrated care for older adults, Activity of daily living, Frailty, Older adults, ICOPES-TW

## Abstract

**Background:**

Intrinsic capacity (IC) is a comprehensive indicator of the overall well-being of older adults, and assessing of IC can help identify early stage of disability and tailor intervention to individual needs. However, there is a lack of effective and simple IC assessment tools. This study aimed to establish predictive scoring algorithms of IC to identify older adults at high risk of impaired functional ability.

**Methods:**

We conducted a cross-sectional study in Southern Taiwan, measuring IC using 7 subitems: cognition, locomotion, vitality, vision, hearing, psychological well-being, and medication usage were measured. Functional ability outcomes included frailty, basic activities of daily living, and instrumental activities of daily living (IADL). The capability of 7 domains of IC in predicting functional ability was assessed by multivariable logistic regression. The prediction of capability of scoring algorithms was indicated by receiver operating characteristic (AUC) curves and measures of sensitivity and specificity.

**Results:**

A total of 1,152 older adults were recruited and analyzed. Locomotion emerged as a significant predictor of IADL disability and worsening frailty. The IC-based weighted scoring algorism for predicting IADL demonstrated satisfactory capability (AUC: 0.80), as did the algorithm for predicting worsening frailty (AUC: 0.90). The optimal cutoff points for predicting IADL disability and frailty worse were estimated respectively at 13 and 16, with sensitivity/specificity values of 0.74/0.75 for the IADL prediction algorithm and 0.92/0.77 for the frailty prediction algorithm.

**Conclusion:**

Our 7-domain IC screening tool proves to be sensitive and practical for early identification of functional disability and frailty among community-dwelling older adults in Taiwan.

## Introduction

The world is undergoing a significant demographic shift toward an aging society. According to the World Health Organization (WHO), by 2030, one in six individuals worldwide will be aged 60 years or older, with the global population of older adults reaching 1.4 billion [1]. Consequently, addressing functional decline and promoting the overall well-being of older adults have become crucial issues worldwide [2]. In 2015, the WHO released the World Report on Ageing and Health, defining intrinsic capacity (IC) as “the amalgamation of an individual’s physical and mental capacities, as well as their interaction with relevant environmental factors, which ultimately determine the person’s functional ability throughout their life course” and highlighting its significance for healthy aging [3]. To operationalize the concept of IC more effectively, the WHO developed the Integrated Care for Older People (ICOPE) model, a multidimensional approach aimed at comprehensive assessment of IC [4]. ICOPE focuses on six key domains of IC, namely cognition, mobility, vitality (including nutrition and mood), vision, and hearing [3]. The primary challenge in IC lies in identifying declines in associated functional abilities before an individual becomes frail, with the goal of delaying or even reversing such decline [5].

Many previous studies have reported the association between IC and functional ability [4, 6–10]. Some studies have even proposed the mediating role that exists between IC and both basic activities of daily living (BADL) and instrumental activities of daily living (IADL) [4, 8, 9]. Recent evidence has also revealed the longitudinal relationship between IC and physical fitness or frailty [11–15]. However, a literature review has pointed out a flaw in the current analysis of ICOPE, highlighting the lack of integration among each domain of IC, as they are often examined separately, and the absence of a standardized summary score for the overall construct of the ICOPE screening tool. This limitation hampers international comparisons and makes it difficult to evaluate IC measures across different target populations [16]. To the best of our knowledge, most studies have treated ICOPE screening tools as individual domains or scored each IC domain separately, without reaching a consensus on a standardized scoring system for the entire ICOPE screening tool [4, 6, 17–21].

In order to identify early loss of functional domains and assess healthcare needs, researchers and policymakers in geriatric care are actively developing IC screening tools for older adults based on the ICOPE framework [6, 15, 22, 23]. However, there is currently a lack of standardized checklist items for ICOPE screening tools across different countries. To address this gap, the Taiwanese government has developed an IC screening tool, called the Integrated Care for Older People Screening Tool for Taiwanese (ICOPES-TW), which incorporates seven domains: cognition, locomotion, vitality, vision, hearing, psychological well-being, and medication usage, in line with the ICOPE framework [24]. Initial psychometric properties of the ICOPES-TW have been tested with satisfactory validity (i.e., good correlations with BADL, IADL, quality of life, and frailty); however, further evaluation of its effectiveness has yet to be conducted [Chen HY, Su HJ, Liu CH, Yang YC, Lin CY. Integrated care for older people screening tool for Taiwanese (ICOPES-TW): a useful screening to assess health for older people. The International Association of Gerontology and Geriatrics Asia/Oceania Regional Congress 2023 June (IAGG Asia/Oceania Regional 2023), Yokohama, Japan. (Poster).]. Therefore, our study aims to construct an optimal scoring algorithm based on ICOPES-TW to predict functional ability (including BADL and IADL) and frailty among community-dwelling older adults in Taiwan.

## Methods

### Participants and data collection

The cross-sectional study was conducted from April 2022 to November 2022, recruiting eligible participants from the community of Tainan City and outpatient clinics at National Cheng Kung University Hospital (NCKUH) in Taiwan. The inclusion criteria were older adults aged 60 years or above, capable of communicating in Mandarin Chinese or Taiwanese, and able to provide informed consent. We excluded individuals who had difficulty understanding the study questionnaires or were severely ill based on medical records. After obtaining approval from the NCKUH Institutional Review Board (IRB No.: A-ER-110-249), trained interviewers explained the research purpose, assisted participants in completing questionnaires, and observed and assessed their condition and performance. The study procedures followed the principles of the Helsinki Declaration, and all participants provided written informed consent. Design and conduct of this study have followed the Strengthening the Reporting of Observation Studies in Epidemiology (STROBE) guideline.

### Measurement

**IC.** IC was assessed using the ICOPES-TW. This seven-subscale questionnaire evaluates IC based on the WHO ICOPE framework [3]. Each domain of IC defined by the WHO, including cognition, locomotion, vitality, vision, hearing, psychological well-being, and medication usage, is represented as a subscale in the ICOPES-TW. The cognition subscale comprised three items (time orientation, location orientation, and a 3-item recall memory test); the locomotion subscale included one item (mobility); the vitality subscale included two items (weight loss over 3 kg and loss of appetite); the vision subscale included one item (difficulty in watching); the hearing subscale included one item (ability to repeat the numbers 6, 1, and 9); the psychological subscale included two items (feeling bothersome and reducing engagement in activities); and the medication subscale included three items (taking 10 or more different medications, taking painkillers or sleeping tablets, and experiencing changes in balance, sleepiness, dizziness, low blood pressure, or dry mouth due to medication). For this study, we focused on the first medication item (taking 10 or more different medications) to represent the medication domain. The ICOPES-TW has been validated among Taiwanese older adults, demonstrating satisfactory psychometric properties, and a lower ICOPES-TW score indicates a higher level of IC ([Lin Chung-Ying, et al.: Development of an IC-based simple scoring algorithm to identify community-dwelling older adults with impaired functional ability in Taiwan (submitted)]). In our study, each domain of IC was scored a 0–1 range to stratify the status of functional impairment (0 = preserved; 1 = impairment). Therefore, the range of total IC impairments score was from 0 to 7.

**Basic activity of daily living (BADL).** BADL, also known as the Barthel Index (BI), is a 10-item questionnaire rated on a Likert-type scale with two-point, three-point, or four-point options. Higher scores indicate better BADL performance. The sum of item scores reflects the overall BADL performance, ranging from 0 (indicating total dependence) to 100 (indicating total independence) [25]. BADL disability was defined as a BADL score < 100. The Chinese version of the BADL has been validated among Taiwanese older adults, demonstrating adequate internal consistency (α = 0.930) [26].

**Lawton Instrumental Activities of Daily Living Scale (IADL).** The Lawton IADL is an 8-item self-reported questionnaire used to assess IADL performance in older adults. Each item is rated on a dichotomous scale, indicating independence (scored as 0) or dependence (scored as 1). A higher score indicates poorer IADL performance. The sum of item scores generates an overall IADL score, ranging from 0 (indicating total independence) to 8 (indicating total dependence) [27]. The Chinese version of the IADL has been validated, demonstrating excellent internal consistency (α = 0.954) [28]. IADL score below 8 was considered as IADL disability in this study.

**Frailty.** Frailty status was assessed using the validated Chinese version of the Clinical Frailty Scale (CFS) [29], which includes nine levels ranging from 1 to 9. Higher CFS scores indicate worse frailty in older adults [30]. We categorized CFS scores of 4–9 as indicative of frailty worse.

### Covariate

We considered several sociodemographic factors of older adults based on previous studies [31, 32]. These factors included age, gender, living arrangement (living alone or with others), education status (uneducated, primary school, junior high school, senior high school or above), and financial independence.

### Statistical analysis

Categorical variables were presented as numbers and percentages, while continuous variables were expressed as means, standard deviation, and range. Differences between characteristics of older adults and the prevalence of BADL disability, IADL disability, and frailty worse were assessed using the chi-squared test. We used logistic regression to assess the association between IC subitems and three outcome measures: BADL disability, IADL disability, and frailty worsening. Adjusted odds ratios (AORs) and their corresponding 95% confidence intervals (CIs) for each outcome were estimated while adjusting for selected covariates.

To develop a scoring algorithm, we assigned weighting and non-weighting to evaluate the prediction model based on significant predictors of IC domain impairment positively associated with functional disability or frailty identified through regression analysis. According to the method suggested by Moons et al. [33], the regression coefficients from the multiple logistic regression multiplied by 10 and rounding to the nearest integer were converted into weight values (i.e., score) for each significant IC subitem impairment. The total score for each patient was summed from the score of each IC subitem. A score of 0 points indicated a non-significant association between the variable and functional disability or frailty. Based on the scoring algorithm, a total score for predicting IADL and frailty ranged 0–55 and 0–72, respectively. The non-weighting score was giving + 1 point for a factor with a significantly positive association with functional disability or frailty status.

We further examined the prediction capability of the scoring algorithm mentioned above for BADL disability, IADL disability, and frailty worse using receiver operating characteristic (ROC) curves. The prediction capability of the simple scoring algorithms was calculated and compared based on the area under the ROC curve (AUC). Sensitivity, specificity, positive predictive value (PPV), and negative predictive value (NPV) were calculated at different cutoff values to identify the optimal cutoff point for functional outcomes [34]. All statistical analyses were performed using R statistical software (version 4.2.3) and the “pROC” package was used to display and analyze ROC curves.

## Results

A total of 1,152 older adults were recruited from the community and outpatient settings for this study. The sociodemographic characteristics and functional performance of the participants are presented in Table 1. The majority of older individuals were in the age range of 70–79 years (44.4%), female (51.8%), living with others (89.2%), had attained a senior high school education or above (44.9%), and were financially independent (61.3%). The proportion of impairment in each domain of IC was as follows: cognition − 23.3%, locomotion − 27.5%, vitality − 16.8%, vision − 44.6%, hearing − 19.2%, psychological − 16.6%, and medication − 16.9%. The median and average scores of total IC composite impairments number were 1 and 1.6 in our participants. However, based on our study findings found that the subdomain might have different weights to predict functional disability or frailty. The ranges of scoring algorithms for predicting IADL and frailty were 0–17 and 0–28, respectively.

**Table 1:** Characteristics of the study participants (*n* = 1,152)

		Total	
		n (mean)	% (SD)
Total		1,152	100.0
Age (years)			
	60–69	449	39.0
	70–79	512	44.4
	80+	191	16.6
	Range: 60–96	(72.4)	(6.9)
Sex			
	Male	555	48.2
	Female	597	51.8
Live alone			
	Yes	124	10.8
	No	1,028	89.2
Educational status			
	Uneducated	93	8.1
	Primary School	373	32.4
	Junior School	169	14.7
	Senior High School or above	517	44.9
Financial independence.			
	Yes	706	61.3
	No	446	38.7
Impairment in IC domains			
	Cognition	268	23.3
	Locomotion	317	27.5
	Vitality	194	16.8
	Vision	514	44.6
	Hearing	221	19.2
	Psychological	191	16.6
	Medication	80	16.9
TotalTotal IC impairment score	Range: 0–7	(1.6)	(1.4)
BADL score: range 25–100		(97.9)	(8.4)
IADL score: range 1–8		(7.3)	(1.6)
CFS score: range 1–8		(2.5)	(1.3)

IC: Intrinsic Capacity; BADL: basic activity of daily living; IADL: instrumental activity of daily living; CFS: Clinical Frailty Scale

**Fig. 1:**
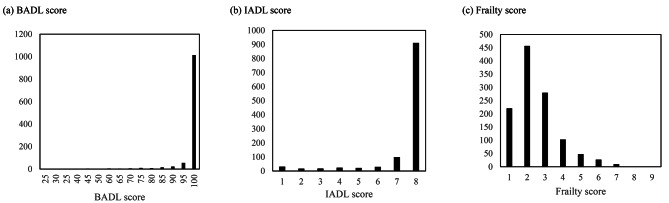
Distributions of score for BADL, IADL, and frailty. BADL: basic activity of daily living; IADL: instrumental activity of daily living

The mean (± standard deviation) scores for BADL, IADL, and CFS were 97.9 (± 8.4), 7.3 (± 1.6), and 2.5 (± 1.3), respectively. Figure 1 indicates that most participants had good performance in both basic and instrumental ADL and were rarely classified as frail.

Table 2 displays the prevalence of impaired BADL, IADL, and frailty among community-dwelling older adults, which were found to be 12.0%, 20.9%, and 16.4%, respectively. Older individuals aged 80 and above, females, those with primary school education, and those who were financially dependent were more likely to have impairment in BADL. However, demographic and sociographic factors were not significantly associated with IADL disability and worsening frailty. Notably, the prevalence of IADL disability and worsening frailty was significantly higher among older adults with impaired IC domains.

**Table 2:** Univariate analyses of various characteristics in association with abnormality of BADL, IADL and frailty worse in older adults

		BADL disability	p value	IADL disability	p value	Frailty worse	p value
		n (%)		n (%)		n (%)	
Total		138 (12.0)		241 (20.9)		189 (16.4)	
Age (years)							
	60–69	28 (20.3)	< 0.001	92 (38.2)	0.737	69 (36.5)	0.329
	70–79	47 (34.1)		112 (46.5)		93 (49.2)	
	80+	63 (45.7)		37 (15.4)		27 (14.3)	
Sex							
	Male	54 (39.1)	0.030	109 (45.2)	0.338	91 (48.1)	1.000
	Female	84 (60.9)		132 (54.8)		98 (51.9)	
Live alone							
	Yes	15 (10.9)	1.000	18 (7.5)	0.082	22 (11.6)	0.767
	No	123 (89.1)		223 (92.5)		167 (88.4)	
Educational status							
	Uneducated	21 (15.2)	< 0.001	21 (8.7)	0.742	17 (9.0)	0.917
	Primary School	59 (42.8)		71 (29.5)		63 (33.3)	
	Junior High School	20 (14.5)		36 (14.9)		28 (14.8)	
	Senior High School or above	38 (27.5)		113 (46.9)		81 (42.9)	
Financial independence							
	Yes	65 (47.1)	< 0.001	148 (61.4)	1.000	122 (64.6)	0.354
	No	73 (52.9)		93 (38.6)		67 (35.4)	
Impairment in IC domain							
	Cognition	34 (24.6)	0.764	93 (38.6)	< 0.001	79 (41.8)	< 0.001
	Locomotion	37 (26.8)	0.923	153 (63.5)	< 0.001	155 (82.0)	< 0.001
	Vitality	18 (13.0)	0.251	80 (33.2)	< 0.001	76 (40.2)	< 0.001
	Vision	62 (44.9)	1.000	137 (56.8)	< 0.001	108 (57.1)	< 0.001
	Hearing	27 (19.6)	0.995	75 (31.1)	< 0.001	72 (38.1)	< 0.001
	Psychological	23 (16.7)	1.000	82 (34.0)	< 0.001	78 (41.3)	< 0.001
	Medication	10 (7.2)	1.000	47 (19.5)	< 0.001	40 (21.2)	< 0.001

IC: Intrinsic Capacity; BADL: basic activity of daily living (disability: <100); IADL: instrumental activity of daily living (disability < 8); Frailty worse: Clinical Frailty Scale > 3)

The sociodemographic factors and the seven domains of IC were entered into a multiple logistic regression analysis, as presented in Table 3. Being aged 80 or above (adjusted OR = 6.59, 95% CI = 3.91–11.12) was significantly associated with BADL disability, but none of the IC domains showed a significant association with BADL disability. On the other hand, each impaired IC domain, except for the vision domain, was significantly associated with IADL disability and frailty. Locomotion was particularly important in predicting both IADL disability and frailty.

**Table 3:** Adjusted odds ratios of abnormality in BADL, IADL, and frailty worse in association with various characteristics among older adults

		BADL disability		IADL disability		Frailty worse	
		AOR	(95% CI)	AOR	(95% CI)	AOR	(95% CI)
Age (years)							
	60–69	ref		ref		ref	
	70–79	1.33	(0.81–2.19)	1.05	(0.72–1.52)	1.30	(0.82–2.06)
	80+	6.59	(3.91–11.12)	1.12	(0.66–1.88)	1.08	(0.56–2.06)
Sex							
	Male	0.70	(0.46–1.05)	0.78	(0.55–1.11)	1.01	(0.66–1.54)
	Female	ref		ref		ref	
Live alone							
	Yes	0.90	(0.48–1.67)	0.52	(0.28–0.96)	1.56	(0.81–3.01)
	No	ref		ref		ref	
Educational status							
	Uneducated	ref		ref		ref	
	Primary School	0.98	(0.53–1.82)	0.94	(0.48–1.84)	1.33	(0.58–3.05)
	Junior High School	0.93	(0.44–1.97)	1.25	(0.58–2.72)	1.40	(0.53–3.71)
	Senior High School or above	0.58	(0.29–1.14)	1.29	(0.65–2.54)	1.06	(0.46–2.48)
Financial independence.							
	Yes	0.74	(0.5–1.09)	0.93	(0.66–1.32)	1.18	(0.76–1.81)
	No	ref		ref		ref	
Impaired IC domain							
	Cognition	1.12	(0.71–1.77)	1.76	(1.22–2.54)	1.56	(1.00-2.42)
	Locomotion	0.99	(0.63–1.57)	5.28	(3.75–7.43)	16.27	(10.47–25.31)
	Vitality	0.62	(0.34–1.13)	1.75	(1.16–2.64)	2.30	(1.43–3.70)
	Vision	1.05	(0.71–1.55)	1.35	(0.97–1.89)	1.09	(0.72–1.65)
	Hearing	1.16	(0.71–1.88)	1.50	(1.02–2.21)	2.40	(1.53–3.78)
	Psychological	1.12	(0.65–1.92)	2.17	(1.44–3.28)	2.92	(1.81–4.71)
	Medication	1.15	(0.54–2.44)	4.17	(2.35–7.40)	3.37	(1.71–6.64)

IC: intrinsic capacity; BADL: basic activity of daily living; IADL: instrumental activity of daily living; AOR: adjusted odds ratio

**Fig. 2:**
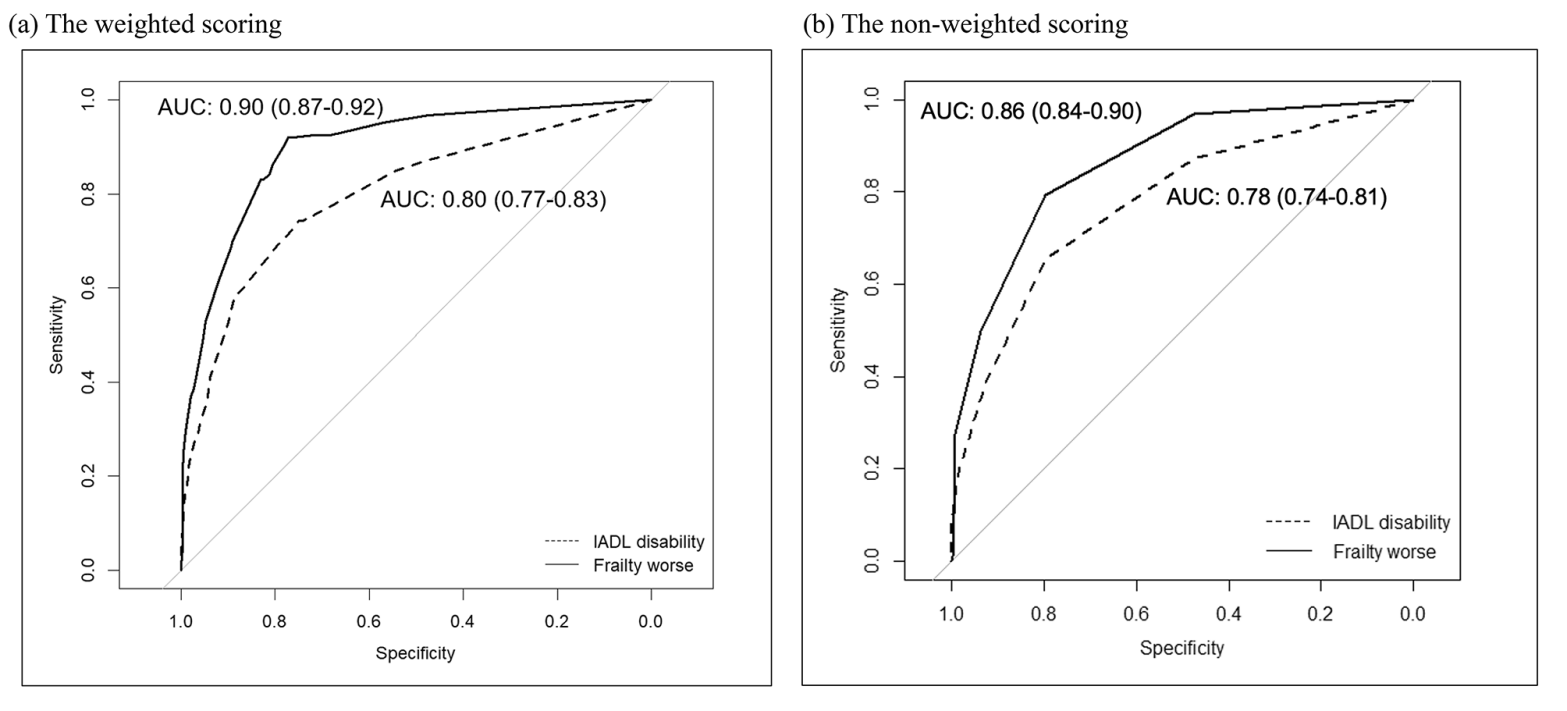
The ROC curves for the scoring algorithm developed in this study for predicting IADL and frailty worse

Since none of the IC domains were associated with BADL disability in the multiple logistic regression analysis, further examination of point score values for predicting BADL disability was not conducted. However, given the significant associations observed between impaired IC domains and IADL disability as well as frailty, a simple scoring algorithm prediction model was developed, respectively and the results are presented in Table 4. The weighted score algorithm generally performed better predictably than a non-weighted score on AUC among IADL (0.80 vs. 0.78) and frailty (0.90 vs. 0.86). Furthermore, the impaired IC domain demonstrated better predictive capability for worsening frailty (AUC = 0.90, 95% CI = 0.87–0.92) compared to predicting IADL disability (AUC = 0.80, 95% CI = 0.77–0.83), as depicted in the corresponding ROC curve shown in Fig. 2. The optimal cutoff points for the weighted and non-weighted risk scores were estimated at 13 and 2 points for predicting IADL disability and 16 and 2 for predicting frailty, respectively. Based on these cutoff points, the weighted scores of sensitivity and specificity for predicting IADL disability were 0.74 and 0.75, respectively, while for predicting worsening frailty, the sensitivity and specificity were 0.92 and 0.77, respectively (Table 4). Additionally, the low positive predictive values (PPV) of 0.44 for IADL and frailty, along with the high negative predictive values (NPV) of 0.92 and 0.98 may indicate a low prevalence of IADL disability or frailty in our study setting. On the other hand, the non-weighted scores in predicting IADL and frailty were 0.66 and 0.79, respectively, for sensitivity, and 0.80 and 0.80, respectively, for specificity. The PPV for IADL and frailty were 0.46 and 0.43, respectively, while the NPV were 0.90 and 0.95, respectively (Table 4).

**Table 4:** Development and performance of the simple scoring algorithm developed in this study for predicting abnormality of IADL and CFS in older adults

	Score for predicting IADL disability				Score for predicting frailty worse			
	AOR	Regression coefficient	Weighting	Non-weighting	AOR	Regression coefficient	Weighting	Non-weighting
Impaired IC domain								
Cognition	1.76	0.56	6	1	1.56	0.44	4	1
Locomotion	5.28	1.66	17	1	16.27	2.79	28	1
Vitality	1.75	0.56	6	1	2.30	0.83	8	1
Vision	1.35	0.30	0	0	1.09	0.09	0	0
Hearing	1.50	0.40	4	1	2.40	0.88	9	1
Psychological	2.17	0.78	8	1	2.92	1.07	11	1
Medication	4.17	1.43	14	1	3.37	1.21	12	1
Total score range			0 − 55	0 − 6			0 − 72	0 − 6
Model performance								
AUC (95% CI)			0.80 (0.77–0.83)	0.78 (0.74–0.81)			0.90 (0.87–0.92)	0.86 (0.84–0.90)
Optimal cut-off point			13	2			16	2
Sensitivity			0.74	0.66			0.92	0.79
Specificity			0.75	0.80			0.77	0.80
PPV			0.44	0.46			0.44	0.43
NPV			0.92	0.90			0.98	0.95

IC: intrinsic capacity; IADL: instrumental activity of daily living; AUC: area under the ROC curve; PPV: positive predict value; NPV: negative predict value; AOR: adjusted odds ratio

## Discussion

This study of healthy ageing concept inspires a new sight for health care in older people to focus on optimizing IC for further prevention the functional disability as they age. In the present study, we used a cross-sectional sample of older community-dwellers to explore the prevalence of impaired IC varied from 16.6 to 44.6% while the proportion of disabled ADL, IADL, and worsening frailty were 12.0 to 16.4%. To the best of our knowledge, this is the first study to survey the develop a model for predicting the functional ability of community-dwelling older adults in Taiwan. The algorithm-based model was well calibrated by integrating 7-domain of ICOPES-TW which was useful for prediction advanced daily function and detection worsening frailty among older adults. In addition, this model exhibited a moderate ability for discriminating between community-dwelling older adults with versus without IADL disability and worsening frailty.

Our study showed that age, gender, living alone, educational status, and financial independence were significantly different between older adults with or without BADL disability, but not in IADL disability or worsening frailty. Our result revealed IC scores independently predicted impairment of IADL and frailty, but for BADL, which is similar as a previous study in China [35]. Consistent with previous literature, the IC can provide valuable predictive information on an individual’s function [36, 37] and frailty status [13, 37], even after accounting for the number of personal characteristics and multimorbidity. However, our result was inconsistent with a structural equation analyses research showed IC had directed and indirect relationships with BADL [36]. The reason might because that our study sample was relative health and independence who were recruited from either outpatient clinics or health check-up community centers. Our study also found personal characteristics (e.g., age, gender, education, and financial independence) could significantly predict BADL disability; but failed to predict IADL disability and worsening frailty in community-dwelling older adults, which is similar as previous studies [38, 39].

In this study, we integrated IC-related variables from the aforementioned WHO conceptual framework [40] to propose a simple scoring algorithm. The scoring system dichotomizes older adults into high-risk (weighted cutoff ≥ 13 for IADL and ≥ 16 for frailty; non-weighted ≥ 2) and low-risk (weighted cutoff < 13 for IADL and < 16 for frailty; non-weighted < 2) populations to maximize the sensitivity and specificity of the prediction of IADL disability and worsening frailty. Therefore, early identification using four or more factors from a total of seven significant IC predictors among older adults can be an effective strategy for future clinical practice. Nonetheless, under the low prevalence of functional disability among the 1152 older adults included in this study, 861 (179 true positive plus 682 true negative) and 918 (174 true positive plus 744 true negative) were correctly functioning predicted with the overall accuracy of 74.7% and 79.7% for IADL disability and frailty worse respectively on the basis of proposed weighted cut-off points. Therefore, 408 and 393 older adults were recommended to undergo further IADL and frailty intervention respectively, although only 179 or 174 actually had IADL disability or frailty worse, resulting in a PPV of 43.9% versus 44.3%. Moreover, the overall accuracy for predicting IADL disability and worsening frailty was 76.6% and 79.7% respectively, based on the proposed non-weighted cutoff points. Out of the 882 older adults correctly predicted to be functioning well, 158 had actual IADL disability and 724 did not. Similarly, out of the 918 older adults correctly predicted to be functioning well, 150 had actual worsening frailty and 768 did not. In total, 345 older adults were recommended for IADL intervention and 210 for frailty intervention. However, only 158 and 150 of them respectively actually had IADL disability or worsening frailty. This results in a PPV of 45.8% for IADL disability and 71.4% for worsening frailty. Given the importance of early identification and strategy provision to prevent functional deterioration among high-risk community-dwelling older adults, our proposed scoring algorithm could be still considered useful in community practice even though several older adults could be over-diagnosed and intervened.

Although both weighted algorithms developed in this study had satisfactory predicted values with the 13 and 16 cut-off points for older adults’ functional ability, our result showed IC better predicted for worsening frailty compared to the IADL disability. Frailty, defined as the progressive decline of physiological systems with increasing vulnerability to stressors and exposing risks of adverse health outcomes, exhibits many similarities as the IC (i.e., composite of all mental and physical capacities) [41]. A Korean study has mentioned IC and frailty represented the two faces of the same coin, with one indicating the reserves of the individual and the other denoting the deficits that accumulate with aging [42]. Therefore, under certain aspects, IC might be considered as a sort of evolution of frailty; both attempts to anticipate the importance and necessity of comprehensively evaluated and adequately managed the aging individual based on integration and multidisciplinary of services [15]. Similarity with previous studies conducted in developing and developed country, our study revealed that older adults with impaired IC reported higher odds of functional disability [43]; as well as the other evidence that showed locomotion (e.g., grip strength and gait speed) could significantly predict IADL disability, but not other evaluated outcomes, such as BADL difficulty and frailty [44].

Furthermore, our study classified the locomotion domain of IC as either impaired or normal based on the time it takes for adults to transition from a seated to a standing position and back five times. If this duration exceeds 12 s, it indicates impaired locomotion in older individuals [45]. Frailty is assessed using CFS, a scale from 1 (very fit) to 9 (terminally ill), which evaluates mobility, energy, physical activity, and overall function based on the interviewer’s clinical judgment of older people [30]. In our study, frailty scores ranging from 4 to 9 were considered as cutoff points for worse frailty. Since a frailty score of four or above indicates that older people often experience limited activity symptoms and commonly complain of “slowing up,” individuals with this issue may have a significantly higher likelihood of failing the evaluation to stand and perform five back positions within 12 s. Due to the commonality of the contents existed within the two measures, our study has a strong association between locomotion impairment and increased frailty. Therefore, we suggested that the locomotion status could impact the progression of frailty.

Our simple scoring algorithms integrating the WHO ICOPE framework IC measures (i.e., cognition, locomotion, vitality, vision, hearing, psychological, and medication) is suitable for determining the most relevant determinants of disability and frailty among community-dwelling older adults. Among the seven IC-related components, six predictors expect vision capacity were identified to be significantly associated with the functional abilities in varied contribution. The results are consistent with a study assessing the performance of diagnostic measures of the ICOPE screening tool in European community-dwelling older adults, which showed only vision, but not other IC-related domains vision, had the lowest sensitivity among all IC domains between normal and altered population [46]. Among all IC predictors in the scoring algorithm, locomotion weighted most in predicting IADL and frailty. Previous studies had similar result; one research revealed impaired locomotion significant predicted frailty in 3 years [47] while the other French study had reported each IC domains demonstrated positive association with the risks of incident frailty and ADL disability, especially the limited mobility played the most critical role with 2.97 hazard ratio to predict worsening frailty [48]. Furthermore, we have identified polypharmacy is the second strong predictor for IADL and frailty among community-dwelling older adults in Taiwan. Several studies have added some relevant components, not only medication [49, 50] but also fall or urinary incontinence [42], for the comprehensiveness of the WHO ICOPE framework measure.

Although several articles investigated a similar prediction question, they were not identical in methodology [43, 47, 48, 51]. Jia et al. [47] conducted the longitudinal cohort study using the 3-year transition (keep well, improved, worsened, and kept poor) of IC and found impaired vitality and locomotion were associated with worsened or kept frail. In contrast, our study is a cross-sectional design. Shen et al. [51] employed a cross-section study design similar to ours, but they defined each domain on a 0–2 score range to represent the functional status to predict frail risk and frailty. They found vision impairment increased the risk of frailty after adjusting for the related potential confounders; however, the presented study showed impaired vision is not a significant predictor for either IADL or frailty and has been excluded from the scoring algorithm. Zeng et al’s study [43] determined the normal as 1 and decline as 0 in each domain and combined the vision and hearing as one score to represent the sensory domain status, which differs from our approach. Multivariate regression models showed only cognition, but no other IC domains showed significance to the 1-year follow-up IADL function. The other study by González-Bautista et al. [48] calculated the total score by adding the number of IC impairments (score range 0–6, with higher scores indicating greater impairment) as a marker of a higher risk of frailty and disability. The results showed limited mobility imposed the highest risk of incident frailty among IC domains over five years, and visual impairment cut points for the ICOPE sum score were ≥ 3 for incident frailty and ≥ 2 for incident IADL; both method and result are very close to our study. However, our novelty is that we used the regression coefficient weighting method to give each domain’s point of risk score. Thus, our study finding provided an evidence-based recommendation to incorporate the medication domain into the ICOPE-TW screening.

This study proposed that both weighting and non-weighting scoring systems yield similar AUCs for IADL (0.80 vs. 0.78) and frailty (0.90 vs. 0.86), indicating that both can be employed. Nonetheless, the scoring algorithm sensitivity in weighting is noticeably higher (0.91 vs. 0.79), resulting in fewer false negatives. This improvement in sensitivity helps identify elderly individuals who truly require subsequent interventions.

Our study has some limitations. First, participants were recruited from residents in southern Taiwan by using the convenience sampling method, potential selection bias restricted the generalizability might exist. The cross-sectional design also restricted our ability to investigate the temporal relationships between IC-based score and IADL and frailty. In terms of making predictive interpretations of our findings, we should be cautious. Second, although we had considered several confounders and adjusted in our prediction models; however, the potential of residual confounding cannot be completely eliminated. Third, for determining functional ability, the frailty measure in this study relied on the single item assessment while both BAAL and IADL assessments were self-reported. Thus, additional multi-item questionnaires or objective measures might be required to recognize functioning to prevent the potential for outcome misclassification bias. In addition, most measures in the present study were based on the nature of self-reports; therefore, reporting bias due to social desirability responding in self-reported measures may exist. Fourth, we evaluate the predictive performance of a model with the same data we used for training. It might be an exaggerated model performance. However, some argue that randomly split sample data into training and test data might ignore autocorrelation in data, still leading to an over-optimistic assessment of model predictive power [52]. Furthermore, the low prevalence rate of IADL disability and frailty (20.9% vs. 16.4%) in our sample may affect the prediction ability (i.e., NPV) when applied in other populations. For example, as for nursing home residents with higher prevalence of functional disability or frailty, there could be higher probability of positive results from ICOPE screening and in fact have functional disability. Therefore, further large-scale study with broader spectrum population such as residents in nursing home and is necessary to increase the utility of this algorithm. Thus, larger population studies with prospective longer-term outcome measures are necessary to validate our study findings.

## Conclusion

We proposed a simple scoring algorithm with substantial sensitivity and satisfactory specificity with fair PPV and high NPV to assess the risk of among community-dwelling older adults. The implementation and utility of this algorithm in the community may not only help clinicians to assess and identify the functional level among older adults but also assist researchers to establish intervention strategies according to impaired IC domains for community-dwelling older adults.

## Data Availability

The datasets generated and/or analyzed during the present study are not publicly available owing to patient privacy and ethical issues. However, they are available from the corresponding author upon reasonable request.
